# Mothers with Dysregulated Eating and Their Offspring’s Emotional/Behavioral Functioning during the COVID-19 Pandemic: A Descriptive Study

**DOI:** 10.3390/jcm13041018

**Published:** 2024-02-09

**Authors:** Luca Cerniglia, Silvia Cimino

**Affiliations:** 1 Faculty of Psychology, International Telematic University Uninettuno, 00186 Rome, Italy; 2 Department of Dynamic, Clinical and Health Psychology, Sapienza University of Rome, 00185 Rome, Italy; silvia.cimino@uniroma1.it

**Keywords:** COVID-19, dysregulated eating, internalizing/externalizing symptoms, longitudinal study

## Abstract

Objective: Research on the psychopathological effects of the COVID-19 pandemic has not specifically focused on mothers with dysregulated eating and their children. Methods: This study aimed to observe whether the symptoms of mothers with binge eating episodes (assessed through the SCL-90/R and the TFEQ-R18) worsened from the pre-pandemic period (T1) to the pandemic period (T2). In addition, we sought to assess whether the levels of internalizing/externalizing and dysregulation symptoms in children (assessed through the CBCL 6–18) worsened from T1 to T2. Results: Our results showed that the values obtained for mothers in the SCL-90/R were significantly higher at T2 (during the pandemic), particularly for Depression, Phobic Anxiety, Interpersonal Sensitivity, and Obsessive–Compulsive subscales. Moreover, in both the Emotional Eating and Uncontrolled Eating subscales of the TFEQ-R18, mothers at T2 scored substantially higher than mothers at T1. The emotional/behavioral functioning of children was more maladaptive at T2, according to mothers, especially for the subscales of Withdrawn, Anxious/Depressed, and Aggressive Behavior. Children also had significantly higher scores on the Internalizing and Externalizing subscales, as well as greater symptoms of dysregulation. Conclusions: This study contributes to demonstrating that the COVID-19 pandemic may have had increased maladaptive emotional/behavioral functioning in children and their mothers with dysregulated eating.

## 1. Introduction

Previous research has widely demonstrated that stressful experiences can be associated with and/or predict emotionally and dysregulated eating [1,2], and the pandemic period of COVID-19 can indeed be considered a stressful situation [3]. COVID-19 is an unknown and extremely contagious disease that has killed five million people globally so far [4]. Since February 2020, SARS-CoV-2 has spread worldwide, affecting people’s lives in a variety of ways, including interpersonal and social interactions, work and school habits, freedom of movement and travel, and family and childcare practices [5]. The previous literature had already posited the possible negative impact of pandemics on parental and offspring mental health due to social distancing and isolation from peers and family [6]. Several research studies have focused on the possible effects of the blockade and quarantine (and the pandemic in general) on adults, adolescents, and children [7,8]; however, little is known about how and in what ways the pandemic has affected psychopathological symptoms and the severity of subthreshold clinical conditions, particularly in parents and mothers [9,10].

A specific group of individuals with eating disorders who may have experienced worsening symptoms during the pandemic comprises those suffering from binge eating episodes (BEEs). While binge eating disorder (BED) constitutes a psychiatric condition with an unusually heavy food intake, no compensatory behaviors, and a subjective experience of loss of control (DSM-5; [11]), individuals with BEEs do not meet the criteria for a psychiatric diagnosis, but present with problematic eating with the same characteristics as BED, albeit with lower symptom severity and less frequent episodes. The little existing research on BEE has demonstrated the important effects of this subclinical condition in parents on the child’s mental health [12]. For example, Reba-Harreleson and colleagues [13] showed that children of mothers who manifested episodes of binge eating were more likely to have depressive or anxious symptoms than those of mothers without BEE [14].

Although the developmental stage around eight years of age is a critical point in determining the future outcomes of children’s psychological well-being [15], there is also a relative paucity of studies focusing on the symptoms potentially manifested by school-age children of mothers with psychopathological risk during the pandemic. Therefore, studies dealing with school-age children and their mothers with psychopathological symptoms are particularly needed. So far, however, to the best of our knowledge, no research has focused on the impact of the COVID-19 pandemic on mothers’ dysregulated eating and its possible consequences in internalizing and externalizing symptoms in their offspring. Indeed, the child’s ability to cope with potentially distressing environmental events (in this case, COVID-19) depends on the parent’s ability to manage this experience, so the child’s response to the pandemic in terms of psychopathological symptoms is highly associated with the parent’s psychological well-being and coping skills [16].

### The Present Study

Given that most of the previous studies have used a cross-sectional research design to provide a snapshot of children’s and adults’ psychopathological symptoms during the pandemic, very few data are available from longitudinal studies to compare pre-pandemic levels of symptomatology in parents and children with the severity of their psychological problems during the pandemic [17]. The present study, with a longitudinal repeated-measures study design, aimed to fill this gap in the literature and to (a) observe whether the symptoms of mothers with dysregulated feeding worsened during the pandemic by comparing their symptomatology levels with pre-pandemic levels and (b) observe whether the levels of internalizing/externalizing and dysregulation symptoms in the school-age offspring of mothers with BEEs worsened during the pandemic period compared with the pre-pandemic period.

## 2. Materials and Methods

### 2.1. Samples and Procedures

Four hundred and sixty-nine (N = 469) mothers and their children were recontacted during the COVID-19 lockdown from a larger sample recruited in Italy for a prior study [18] in the pre-pandemic period (May 2019). The sample was contacted via social media (i.e., Facebook) and via notices posted on online psychology research websites. The inclusion criteria for both mothers and children were as follows: (a) no psychiatric diagnosis in the mothers and/or their children; (b) no medical/physical problem reported in the subjects at the time of recruitment; (c) no medical and/or psychological treatment sought (we chose not to include subjects following a psychological treatment because it would be impossible to discern the role of this intervention on the observed symptoms. Moreover, different subjects could follow different treatments, and this could have been a confounding factor); (d) no COVID-19 current contagion in any family member and no death of any close relative associated with COVID-19 both at T1 (pre-pandemic) and T2 (during the pandemic). From the larger sample described in a previous study [18], only mothers who reported binge eating episodes in the pre-COVID period were selected. It is important to note, however, that none of these mothers was diagnosed with binge eating disorder or any other psychiatric condition. Most of the subjects were Caucasian (96%), 90.2% were from intact family groups, and 75% of the children were firstborns. All mothers filled out an ad hoc socio-demographic questionnaire, and Table 1 shows the characteristics of the sample at T1 (pre-pandemic) and T2 (during the pandemic). The study suffered no attrition from T1 to T2. In the socio-demographic questionnaire filled out at T2, no major changes were reported by the mothers (e.g., separation, divorce, COVID-related loss, or increased workload).

Following the Declaration of Helsinki, all contacted subjects consented to participate in the study after being instructed about the aims and methodology of the study and signed the written informant consent form. The Ethical Committee of Sapienza University approved the current study before it began (n. 0000809-2020). All measures were administered remotely through a web-based survey platform.

### 2.2. Measures

Dysregulated eating behavior in mothers was assessed through the Three-Factor Eating Questionnaire-R18 (TFEQ-R18) [19], which is a revised and shortened version of the original TFEQ [20] with stronger psychometric quality; the TFEQ-R18 is divided into three subscales: Cognitive Restraint, Uncontrolled Eating, and Emotional Eating [19]. For the aims of this study, we chose to use the Emotional Eating (i.e., propensity to overeat in reaction to unpleasant emotions) and Uncontrolled Eating (i.e., propensity to overeat due to a sense of being out of control) subscales. On a four-point scale, higher scores indicate more dysregulated eating behaviors. The response options were: definitely false, mostly false, mostly true, and definitely true. For each participant, average scores were computed for the nine questions assessing uncontrollable eating (e.g., “Sometimes when I start eating, I just can’t seem to stop”) and the three questions assessing emotional eating (e.g., “When I feel blue, I often overeat”). Higher scores on the respective scales are indicative of greater uncontrolled or emotional eating (mean scores were used here). In this study, Cronbach’s alpha was 0.87 for uncontrolled eating and 0.89 for emotional eating, indicating excellent reliability.

Psychopathological symptoms in mothers were assessed using the SCL-90/R 1 [21], a 90-item self-report symptom inventory measuring psychopathological symptoms and psychological distress rated on a Likert scale from 0 (not at all) to 4 (extremely). It asks subjects to report whether they have suffered in the past week from symptoms of somatization (e.g., headaches), obsessive compulsivity (e.g., having to check and double-check what you do), interpersonal sensitivity (e.g., feeling that people are unfriendly or dislike you), depression (e.g., feeling blue), anxiety (e.g., feeling fearful), hostility (e.g., having urges to beat, injure, or harm someone), phobic anxiety (e.g., feeling afraid to go out of your house alone), paranoid ideation (e.g., persecutory beliefs concerning a perceived threat toward oneself), and psychoticism (e.g., having thoughts that are not your own). Aside from these nine primary scales, the questionnaire provides a global severity index (GSI). In this study, the SCL-90/R showed a high internal consistency (Cronbach’s alpha = 0.88).

Internalizing and Externalizing problems in children were assessed through the Child Behavior Checklist, version 6–18 (CBCL; see [22]) (Italian validated version; see [23]). This tool is a self-administered questionnaire containing 118 items. Mothers respond to the items on a three-point scale (0 = not true, as far as you know, 1 = somewhat or sometimes true, or 2 = very true or often true) based on the past 6 months. The checklist measures eight empirically based syndromes: Anxious/Depressed (example item: “Fears going to school”), Withdrawn/ Depressed (example item: “Too shy or timid”), Somatic Complaints (example item: “Nausea, feels seek”), Social Problems (example item: “Clings to adults or is too dependent”), Thought Problems (example item: “Hears sounds or voices that aren’t there”), Attention Problems (example item: “Can’t concentrate, can’t pay attention for long”), Rule-Breaking Behavior and Aggressive Behavior (example item: “Breaks rules at home, school, or elsewhere”), and three broad-band scales (Internalizing, Externalizing, and Total Problems). Moreover, the Dysregulated Profile is an aggregate of the Anxious/Depressed, Aggressive Behavior, and Attention Problems subscales [24]. In this study, Cronbach’s alpha value was 0.89.

### 2.3. Data Analyses

Since this is a study with a longitudinal repeated-measures study design, we chose to use analysis of variance (ANOVA) for repeated measures to compare results for all variables at T1 and T2. Repeated-measures ANOVA approaches have traditionally been used to analyze longitudinal data [25,26,27]. However, ANOVA only allows the comparison of averages, sacrificing individual data. ANOVA seemed to be the best choice for two reasons: first, because the present study did not intend to analyze subject-specific trends but to compare group averages of variables measured at T1 and T2, and second, because there were no missing data since the number of subjects remained the same between T1 and T2. The computed *p* values are presented, with values less than 0.05 considered significant. The standard deviations (SDs) are included in the mean values.

We chose to use eta-squared (η^2^) to measure the effect size between variables, one of the most widely used effect sizes in psychological research: according to Lakens [28], when reporting effect sizes for ANOVAs, it is recommended that the generalized eta square is reported instead of the partial eta square. The correlation ratio, η (eta), measures the degree of association between two variables. The square of the correlation ratio, η^2^, is the differentiation ratio; it measures the proportion of the variance of the dependent variable that is associated with the independent variable. By definition, its value varies between 0 and 1: it takes the value 0 when the independent variable explains no variance of the dependent variable, while it takes the value 1 when the independent variable in question explains all the variance of the dependent variable [29].

According to Cohen’s recommendations [30], the power was set at 0.05, and a power of 0.854 was attained with a large effect size of (f2 = 0.43). We used IBM SPSS v.26 to conduct the data analysis.

## 3. Results

For mothers, an ANOVA of the SCL-90/R subscale scores indicated a significant main effect of time point (*p* < 0.001). At T2, SCL-90/R values were substantially higher; mothers, in particular, had high scores for Depression, Phobic Anxiety, Interpersonal Sensitivity, and Obsessive–Compulsive subscales. At the TFEQ-R18, mothers at T2 scored significantly higher than at T1 in both the Emotional Eating and Uncontrolled Eating subscales. The means and η^2^ values are shown in Table 2.

Children’s emotional/behavioral functioning was rated by mothers as more maladaptive at T2, especially for the subscales of Withdrawn, Anxious/Depressed, and Aggressive Behavior. Children also showed significantly higher scores for the Internalizing and Externalizing subscales and presented higher levels of dysregulation problems. Table 3 shows the means and η^2^ values.

## 4. Discussion

This study aimed to observe whether the symptoms in mothers with binge eating episodes intensified during the pandemic by comparing their levels of psychopathology with pre-pandemic levels. In addition, this study aimed to assess whether the levels of internalizing/externalizing and dysregulation symptoms in the children of mothers with BEE worsened from the pre-pandemic to the pandemic period.

Our results showed that SCL-90/R values were significantly higher at T2, with mothers scoring particularly high on the Depression, Phobic Anxiety, Interpersonal Sensitivity, and Obsessive–Compulsive subscales. Moreover, on both the Emotional Eating and Uncontrolled Eating subscales of the TFEQ-R18, mothers at T2 scored substantially higher than mothers at T1. These results are in line with previous research that has shown that the COVID-19 pandemic constitutes a very distressing environmental event that can worsen current mental health in those who were already at risk in the pre-pandemic period [31,32]. Regarding mothers’ dysregulated feeding, although, to our knowledge, this study is the first to specifically assess mothers’ symptoms, this finding is in line with the previous literature that has shown that individuals already suffering from problems in the area of feeding before the pandemic may have experienced a worsening of their symptomatology for a variety of reasons [33]. For instance, lockdowns during the COVID-19 pandemic may have caused an increase in the number of meals and snacking [34]; in addition, individuals with disordered eating frequently report a high level of intolerance to uncertainty [35,36], making them particularly prone to increases in discomfort as a result of the ambiguity and unpredictability that characterized the pandemic period. Significant changes in habits that may result in changes in body shape or weight may further exacerbate eating-related anxiety in this population [37,38]. It is noteworthy that mothers seem to have suffered an increase in their dysfunctional symptoms both in the area of impulse dysregulation and hyperarousal (with binge eating episodes) and in the area of mood symptoms (depression and anxiety). It has been hypothesized that the COVID-19 pandemic period acted as a powerful nonspecific amplifier of psychopathological risk [3]. This could be due to the fact that the pandemic period in itself has been characterized by forced periods of stay-at-home inactivity (which could have fostered withdrawal and depression symptoms) and moments of severe anxiety connected with the dramatic physical risk of the disease, which was communicated by information agencies several times daily. According to our results, as mentioned above, mothers also showed higher interpersonal sensitivity. This result could be related to the nature of the pandemic outbreak itself and the unprecedented measures taken to contain it, which significantly impacted social interaction routines and habits. Very quickly, individuals were asked to distance themselves and maintain a vigilant attitude toward other people; this may have increased the subjects’ vigilance over the actions and intentions of others, thereby increasing interpersonal sensitivity. Regarding all these increased symptoms, however, other studies should verify whether they are acute clinical manifestations, or whether they will persist over time.

Parental psychopathological symptoms are commonly related to children’s maladaptive outcomes, particularly internalizing/externalizing and dysregulation problems, according to the Developmental Psychopathology conceptual model [39,40]. In addition, mothers’ dysregulated feeding has been proposed as a determinant in child development with a key role in modulating offspring’s mental health [41,42]. Indeed, the second hypothesis of the present study predicted that an increase in psychopathological symptoms in mothers was associated with an intensification of the child’s problems. In fact, at T2, mothers reported that children’s emotional/behavioral functioning was more maladaptive, particularly for the subscales of Withdrawn, Anxious/Depressed, and Aggressive Behavior. Children also scored considerably higher on the Internalizing and Externalizing subscales, as well as presenting more dysregulation symptoms. Our findings suggest that no school attendance, frequent confinement at home, and no sharing of any social context with peers may have played a part in this outcome [43,44]. In fact, children of this age (eight/nine years) experiment with their emotion regulation capacities in environments shared with peers, where they are usually learning a balance between the unorganized vital spur and focused, less impulsive activities [45,46,47,48]. The pandemic disrupted these possibilities due to frequent lockdowns and social distancing. The fact that mothers remained almost constantly at home with dysregulated feeding may have exacerbated children’s symptoms, as it has been hypothesized that individuals with BEEs exhibit high levels of autonomic arousal and negative affect that may have led to more intrusive or withdrawn parenting, which in turn may have resulted in higher psychopathological risk in children [49]. These results may be further due to the fact that children may have been exposed to stressful environments, and the family as a whole may have lacked social support [50,51]. During the pandemic, and particularly during the lockdown, social support and interactions between people were inevitably and severely hampered, and the stressful situation may have prevented parents from the maintaining of sufficiently attentive and receptive family relationships [52,53,54], which has been widely shown to be a critical protective factor [55,56].

### Limitations

For the present study, we chose a descriptive design: although longitudinal, it was not possible to investigate causal effects among the variables considered, since it was a repeated-measures research design. The first limitation of this study is that it is limited to comparing the situation before and during the pandemic. The second limitation is that we did not focus on fathers. Thirdly, it has not been confirmed whether the questionnaires were completed by children rather than their mothers. Fourth, the time-responses have not been evaluated. Moreover, this study did not focus on the possible effects of COVID-19 in families, looking at siblings [57].

Finally, the mothers’ and children’s psychopathological symptoms were assessed through self-report questionnaires, and more objective assessment procedures are needed before any firm conclusions can be drawn about these results; in particular, the increase in problems in children might be influenced by the increase in symptoms in mothers, which might have impaired their judgment. However, these instruments are widely validated and widely used in this field, even in studies of subjects with psychopathology [58], confirming the robustness of the data.

Future studies should test the robustness of our findings to understand the relationship between the variables considered and to establish their causality, rather than being limited to a descriptive design. Furthermore, since previous research has shown that fathers can moderate or mediate the effect of mothers’ psychopathology on offspring outcomes [59,60,61], future research should also consider the role of fathers. Finally, in order to obtain objective assessments, and consequently more valid results, it would be appropriate to limit the use of self-report instruments and to use measures such as interviews instead.

## 5. Conclusions

The present study observed that when comparing the levels of psychopathology before and during the COVID-19 pandemic, the symptoms of mothers with binge eating episodes worsened and intensified during the pandemic. In addition to this, the study explored dysregulation and internalizing and externalizing problems in the children of these mothers; again, the results obtained show that both the levels of internalizing and externalizing symptoms and dysregulation in these children worsened during the pandemic compared to the pre-COVID-19 measurement.

Thus, the COVID-19 pandemic, with its devastating effects (both material and affective), affected the emotional/behavioral functioning of both mothers and offspring, as, by increasing the psychological difficulties of mothers, it consequently hindered and/or depleted children’s self-regulation.

## Figures and Tables

**Table 1:** Demographics.

	T1	T2	N_tot_
Children’s gender	222 M, 247 F	222 M, 247 F	469
Children’s age, M (SD)	8.3 (0.18)	9.21 (1.12)	
Mothers’ age, M (SD)	33.25 (2.52)	34.65 (2.41)	
Household income	Approx. EUR 2500/month	Approx. EUR 2500/month	
Educational level	At least 12 years of schooling	At least 12 years of schooling	

M = male; F = female.

**Table 2:** Means (standard deviation) and η^2^ of the mothers’ SCL-90/R and TFEQ-R18 subscales measured at T1 and T2.

	T1	T2	
	M (SD)	M (SD)	η^2^
SOM	0.27 (0.31)	0.43 (0.36)	0.14
**O-C**	**0.45 (0.44)**	1.79 (0.55) **	**0.62**
I-S	0.26 (0.38)	0.98 (0.13)	0.18
**DEP**	**0.58 (0.53)**	1.23 (0.43) **	**0.83**
ANX	0.65 (0.52)	0.77 (0.73)	0.16
HOS	0.29 (0.35)	0.39 (0.27)	0.15
**PHOB**	**0.42 (0.58)**	1.22 (0.28) **	**0.68**
PAR	0.18 (0.55)	0.25 (0.34)	0.17
PSY	0.28 (0.56)	0.22 (0.48)	0.19
**EE**	**22.51 (2.11)**	30.72 (1.25) **	**0.71**
**UE**	**30.12 (0.26)**	36.22 (2.21) **	**0.78**

Note: SOM: Somatization; O-C: Obsessive–Compulsive; I-S: Interpersonal Sensitivity; DEP: Depression; ANX: Anxiety; HOS: Hostility; PHOB: Phobic Anxiety; PAR: Paranoid Ideation; PSY: Psychoticism; EE: Emotional Eating; UE: Uncontrolled Eating. η^2^: eta-squared. Bold lines are significant. ** *p* < 0.001.

**Table 3:** Means (standard deviation) and η^2^ of the children’s CBCL subscales measured at T1 and T2.

	T1	T2	η^2^
**E-R**	**2.53 (1.49)**	**4.62 (2.17)**	**0.68**
**A-D**	**2.21 (1.14)**	5.44 (1.71) **	**0.76**
S-C	2.21 (1.47)	2.32 (2.18)	0.17
WIT	2.77 (1.25)	3.02 (1.85) **	0.14
A-P	1.82 (1.37)	2.07 (1.22)	0.21
**A-B**	**5.14 (2.14)**	8.12 (1.47) **	**0.78**
**INT**	**10.31 (2.45)**	19.21 (3.12) **	**0.75**
**EXT**	**11.41 (2.48)**	16.53 (2.78) **	**0.78**
**DYS**	**6.25 (1.32)**	9.58 (2.11) **	**0.72**

Note: E-R: Emotionally Reactive; A-D: Anxious/Depressed; S-C: Somatic Complaints; WIT: Withdrawn; A-P: Attention Problems; A-B: Aggressive Behavior; INT: Internalizing Problems; EXT: Externalizing Problems; DYS: Dysregulated Profile. Bold lines are significant. η^2^: eta-squared. ** *p* < 0.001.

## Data Availability

Data will be available on reasonable request to the authors.
